# Use of melatonin plus citrulline or arginine to mitigate ergot alkaloid-induced vasoconstriction in sheep^☆^

**DOI:** 10.1016/j.anifeedsci.2025.116502

**Published:** 2025-09-17

**Authors:** James L. Klotz, Celina M. Checura, John B. May, Susan K. Duckett

**Affiliations:** a USDA-ARS, Forage-Animal Production Research Unit, Lexington, KY 40546-0091, United States; b Piedmont Research and Education Center, Clemson University, Clemson, SC 29634, United States; c Department of Animal and Veterinary Science, Clemson University, Clemson, SC 29634-0311, United States; d Department of Plant and Soil Sciences, University of Kentucky, Lexington, KY, United States

**Keywords:** Arginine, Citrulline, Ergot alkaloids, Melatonin, Sheep, Vasoconstriction

## Abstract

Fescue toxicosis, a form of ergotism, causes chronic vasoconstriction in livestock that negatively impact livestock performance. Melatonin, citrulline, and arginine have all been previously associated with vasodilation. The objective of this experiment was to evaluate dosing of melatonin, melatonin + citrulline, and melatonin + arginine in sheep as potential supplements to offset the vasoconstriction caused by toxic tall fescue seed (E + ). Eighteen Suffolk lambs were randomly divided across 3 treatments: melatonin (MEL), MEL+citrulline drench (MEL-CIT) and MEL+arginine drench (MEL-ARG). Jugular blood samples and Doppler ultrasound measurements of carotid artery were taken daily for 12 days. Lambs only received a total mixed ration (TMR) on d 1–3, on d 4–6, lambs received TMR plus E + seed and on d 7–12 lambs received MEL, MEL+CIT, or MEL+ARG. For all 3 treatments, supplementation of MEL resulted in large increases in plasma melatonin on d 7–12 compared to d 1–6 (P < 0.05). The MEL+CIT resulted in 2- to 3-fold increase in plasma citrulline compared to the other 2 treatment groups (P < 0.05). Plasma arginine was greater in MEL+CIT and MET+ARG lambs on d 7 and 8 than MEL (P < 0.05). There was a steady decline in heart rate and luminal area associated across days with initiation of E + seed dosing (P < 0.01) and did not differ across treatment groups. Conversely, velocity increased with E + seed dosing (P < 0.01) and did not differ when treatments were administered. There was no significant effect of treatment on carotid artery blood flow. Increases in plasma melatonin, arginine, or citrulline did not offset vasoconstriction associated with ergot alkaloids.

## 1. Introduction

Ergot alkaloids are mycotoxins that can be found in infested grasses and contaminated small grains and can result in ergotism when consumed by livestock (Klotz, 2015). This malady is comprised of numerous symptoms that result from the ability of ergot alkaloids to interact with biogenic amine receptors (Berde, 1980). One of the outcomes of ergot alkaloid exposure is a sustained vasoconstriction that contributes to reproductive-, gastrointestinal-, and thermoregulatory-related problems associated with ergotism (Strickland et al., 2011). Thus, mitigation of ergot alkaloid-induced vasoconstriction would alleviate many of the problems associated with ergotism.

Oral melatonin supplementation has been shown to increase ovine umbilical blood flow (Lemley et al., 2012; Lemley and Vonnahme, 2017), bovine uterine blood flow (Contreras-Correa et al., 2021), vasodilation of rat mesenteric arteries (Zhao et al., 2017) and has been shown to prevent vasoconstriction in cerebral arteries of fetal sheep (Das et al., 2008). Intravenous supplementation of arginine has resulted in systemic vasodilation and increasing circulating levels of arginine has been shown to result in vasodilation in both humans and sheep (Lorente et al., 1993; Bode-Boger et al., 1998). Oral supplementation of arginine has an added challenge of catabolism by gut bacteria in ruminants (Chalupa, 1976). Conversely, citrulline has been shown to avoid rumen microbial breakdown in both cattle (Gilbreath et al., 2020b) and sheep (Gilbreath et al., 2020a). Citrulline can be effectively converted to arginine in the blood of ruminants (Lassala et al., 2009).

The vasorelaxation that occurs through melatonin supplementation is through the nitric oxide synthase (NOS) pathway and melatonin receptors (MR) MR1 and MR2. Similarly the vasorelaxation that is a result of arginine supplementation is also achieved through the production of nitric oxide. These pathways differ from each other in how they achieve vasorelaxation and different than how ergot alkaloids cause vasoconstriction. This may be a route to achieve mitigation. The objective of this study was to determine if melatonin, melatonin + citrulline, or melatonin + arginine supplementation could mitigate the vasoconstriction associated with consumption of ergot alkaloids in sheep.

## 2. Materials and methods

All experimental protocols involving animals were approved prior to initiation by the Clemson University Institutional Animal Care and Use Committee (AUP2022–0472) and all animal research was completed at the Clemson University Small Ruminant Facility.

### 2.1. Experiment 1 experimental design

A preliminary experiment was conducted to confirm that enteral supplementation of melatonin would result in an elevation of blood melatonin in sheep. Suffolk ewe lambs (n = 10; 8 mo of age; 57.6 ± 3.9 kg BW) were obtained from the Clemson University Sheep Farm. Melatonin (Bulk Supplements; Las Vegas, NV) was dissolved in 95 % ethanol and then applied to a small portion of cracked corn (0.11 kg) and left to evaporate overnight at room temperature without exposure to light (Lemley et al., 2012; Trotta et al., 2021). Ewe lambs were individually fed the cracked corn with (100 μg/kg BW/d; MEL) or without melatonin (CON) prior to feeding the daily allotment of total mixed ration (TMR; Table 1). The experimental period was 4 d of melatonin treatment and jugular blood samples were collected (EDTA vacutainer tubes) on the fourth day of treatment as a serial collection. Blood sampling was initiated prior to feeding supplement (0 h) and at time 0.5, 1, 2, 3, 4, 5, 6, 8, 10, 12, 16 and 20 h post-supplementation. Blood samples were immediately stored on ice, centrifuged at 2000 x *g* for 20 min at 4°C, and plasma was stored frozen at −80 °C until analysis.

### 2.2. Experiment 2 experimental design

**Fig. 1.**
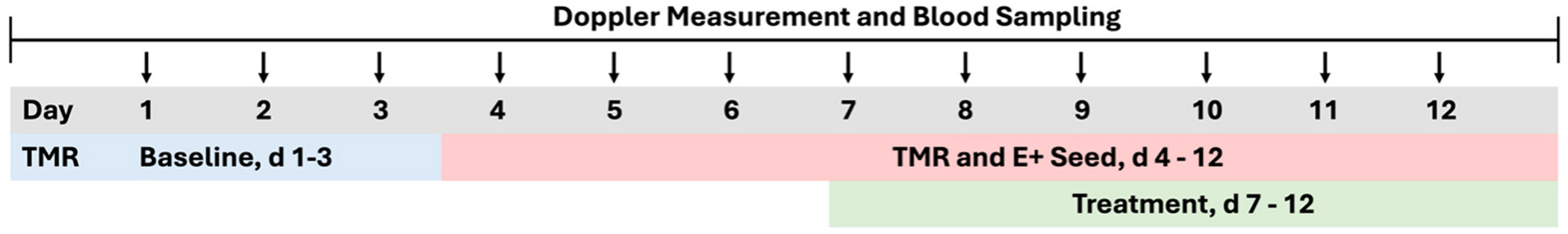
Experimental timeline for Experiment 2. Total mixed ration (TMR) was fed for 3 d as a baseline measurement period. Toxic endophyte-infected tall fescue seed (E + ) was dosed from d 4–12. Lambs were fed TMR daily and melatonin treatment (d 7–12) at 1400. The citrulline and arginine treatments were administered at 0630, 1230, and 1830 daily from d 7–12 on these 2 of 3 melatonin groups. Doppler measurements were collected daily at 1430. Jugular blood samples were taken daily at 1600.

Suffolk ewe lambs (n = 18; 9 months of age; 61.2 ± 4.7 kg BW) were randomly divided into two duplicate groups (n = 9) to facilitate sample collection. In each group, ewe lambs were randomly assigned to one of three treatments (n = 3/treatment/group): 1) melatonin (100 μg/kg BW/d; MEL) fed at 1400 h daily, 2) MEL + citrulline (Bulk Supplements) drench (81 mg/kg BW/dose; 3 doses per day in 8 h intervals; MEL-CIT) and 3) MEL + arginine (Bulk Supplements) drench (81 mg/kg BW/dose; 3 doses per day in 8 h intervals; MEL-ARG). Oral drenches of citrulline or arginine were administered every 8 h based on previous research that showed plasma citrulline to be elevated for 8 h post drenching with citrulline (Greene et al., 2020a). Ewe lambs were individually fed a TMR at 1400 h for 7 d prior to the start of the experiment to become accustomed to feeding stalls and handling. On d -5, lambs were clipped to remove wool in the carotid region for Doppler ultrasound measures. Starting on d 1, cross-sections and blood flow of the left common carotid artery were measured daily at 1430 h (Fig. 1). Ewes were fed TMR only and Doppler measurements were taken daily for 3 d to provide baseline measures. Then ewe lambs were fed TMR plus toxic endophyte-infected tall fescue seed(E + ; 1.77 mg/ewe/d ergovaline + ergovalinine) and daily Doppler ultrasound measurements of cross-sections of left common carotid artery continued at 1430 h on d 4–6 (E + ). Treatments (MEL, MEL-CIT, MEL-ARG) that were evaluated for vasorelaxation were supplemented with TMR and E + fescue seed on d 7–12. Melatonin (100 μg/kg BW) was fed in a small amount of feed as described for Experiment 1 prior to feeding remaining TMR and E + fescue seed. Citrulline-malate and arginine were drenched (81 mg citrulline or arginine/kg BW per dose) at 0630, 1230 and 1830 every day from d 7–12. Ewe lambs were drenched 3 times daily in attempt to keep citrulline and/or arginine concentrations above baseline levels throughout the 24-h period (Greene et al., 2020b). To avoid disrupting tissues for Doppler evaluation, jugular blood samples were collected from the right vein as previously described at 1600 on the same days to measure plasma amino acid levels and melatonin concentrations.

### 2.3. Doppler measurements

For carotid area measurements, the left common carotid artery was imaged in cross-section with the animal standing and nonsedated. A Doppler ultrasound system (LOGIQ^™^ *e* Vet NextGen; GE Healthcare; Wauwatosa, WI) with a 5 – 13 MHz linear transducer (12L-RS) was used to evaluate the artery. At least 3 cine loops were recorded in B mode each day, immediately before the Doppler measurements. Each cine loop was later reviewed; the image with the largest circumference was assumed to be at peak systolic phase and was used to take two diameters perpendicular to each other. The average of the two diameters was used to determine area by using the following equation: area =π (d/2)^2 where d = diameter. The average of 3 areas, one per cine loop, was used for each day.

For Doppler measurements, the transducer was positioned along the longitudinal vessel axis. Three spectral Doppler images, with at least three consecutive representative cardiac cycles each, were obtained per day. The ultrasound software (GE Healthcare) was used to calculate peak systolic (PS), end-diastolic (ED), time-averaged mean velocity (TAMEAN), resistance index (RI), perfusion index (PI), and heart rate (HR) from stored images. Volumetric blood flow (mL/min) was estimated by multiplying cross-sectional area (cm^2^) by time-averaged mean velocity (cm/sec) (Blanco, 2015). Because each animal served as it’s own control, Doppler data are presented as proportionate differences of the response measures (d 4–12) from baseline average (d 1 – 3).

### 2.4. Plasma melatonin and amino acid analyses

#### Melatonin.

Plasma samples from Experiments 1 and 2 were analyzed for melatonin with a method that was originally described by Zhao et al. (2016) and modified as described below. Samples were prepared by combining 1 mL of plasma with 200 μL of water, 200 μL of 1 M sodium carbonate, 5 μL of the internal standard melatonin-d4 (1000 ng/mL; Cayman Chemical Co., Ann Arbor, MI), and 1.5 mL of ethyl acetate. This mixture was placed on a rocker table for 20 min at 50 rpm then centrifuged at 10,000 rpm for 5 min. The organic layer was removed and transferred to an a 2 mL autosampler vial and evaporated to dryness with a centrivap (Labconco Corp, Kansas City, MO) at 35 °C for 45 min (heat turned off after 40 min). Dried samples were reconstituted with 100 μL of initial mobile phase (85 % H_2_O/15 % acetonitrile; both with 0.1 % formic acid). Prepared samples were analyzed via liquid chromatography with a C18 column (Waters Acquity BEH; 2.1 mm×150 mm x 1.7 μm) coupled to a mass spectrometer (LC/MS; Waters Acquity H-Plus UPLC and a Waters Xevo TQ-S Cronos MS; Waters Corporation, Milford, MA).

#### Citrulline and Arginine.

Plasma samples from Experiment 2 were analyzed for citrulline and arginine content as described by Greene et al. (2020b). Plasma samples were spiked with internal standard solution (α-amino butyric acid) and proteins precipitated with the addition of cold methanol followed by centrifugation. The resulting solutions containing plasma amino acids are derivatized with 6-aminoquinolyl-N-hydroxysuccinimidyl carbamate (Waters AccQ-Tag; Waters Corp). Analyte concentrations were determined by LC/MS (Waters Acquity H-Plus UPLC and a Waters Xevo TQ-S-Cronos MS, Waters Corp). Chromatographic separation was obtained using a reverse-phase ultra-performance liquid chromatography (UPLC) column (Waters BEH C18, 2.1 mm×150 mm x 1.7 μm).

### 2.5. Statistical analyses

Data from Experiment 1 were analyzed as a completely randomized design for the effect of melatonin supplementation with SAS (ver. 9.4; SAS Inst. Inc., Cary, NC). For Experiment 2, the ultrasound data were normalized as a proportionate change from baseline mean for each lamb (Aiken et al., 2016). These data and the plasma analyte data were analyzed over day of experiment as a completely randomized design with a repeated measures treatment design using the mixed models of SAS (SAS Inst. Inc). The covariance structure used in repeated statement was autoregressive-1. Lamb was the experimental unit and lamb nested within treatment was the subject. Means separation was done with least significant difference and means were considered different at P ≤ 0.05 and considered as atendency for difference at P ≤ 0.1.

## 3. Results

### 3.1. Plasma data

#### Melatonin.

**Fig. 2.**
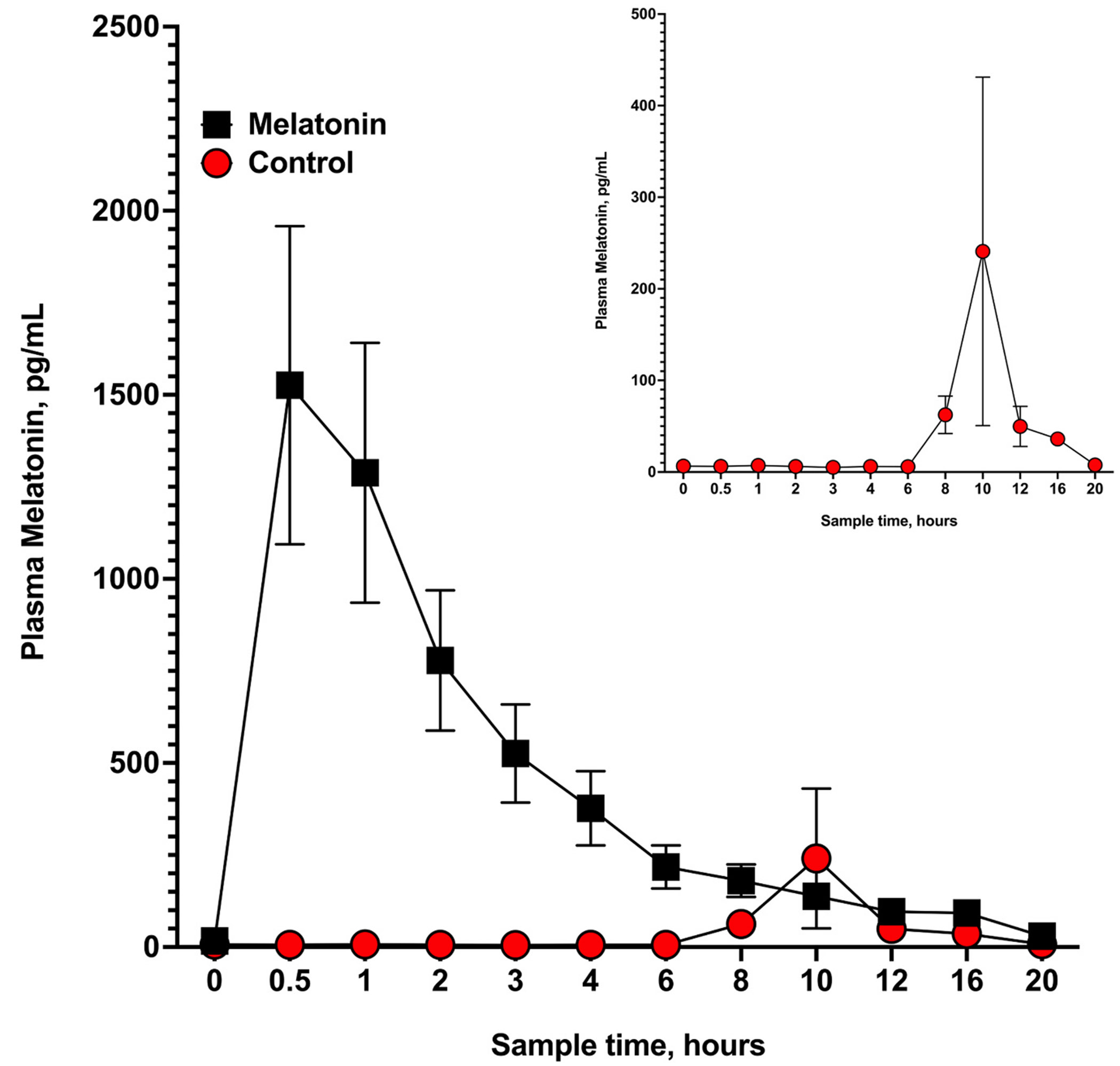
Mean (± SEM) plasma melatonin concentrations over time (0 h = 1400) for Experiment 1 following 4 d of a 100 μg/kg BW/d oral dose. The inset graph is the control lamb data that was less visible with melatonin supplemented lamb data - only to illustrate the scotophase where melatonin levels naturally increase.

**Fig. 3.**
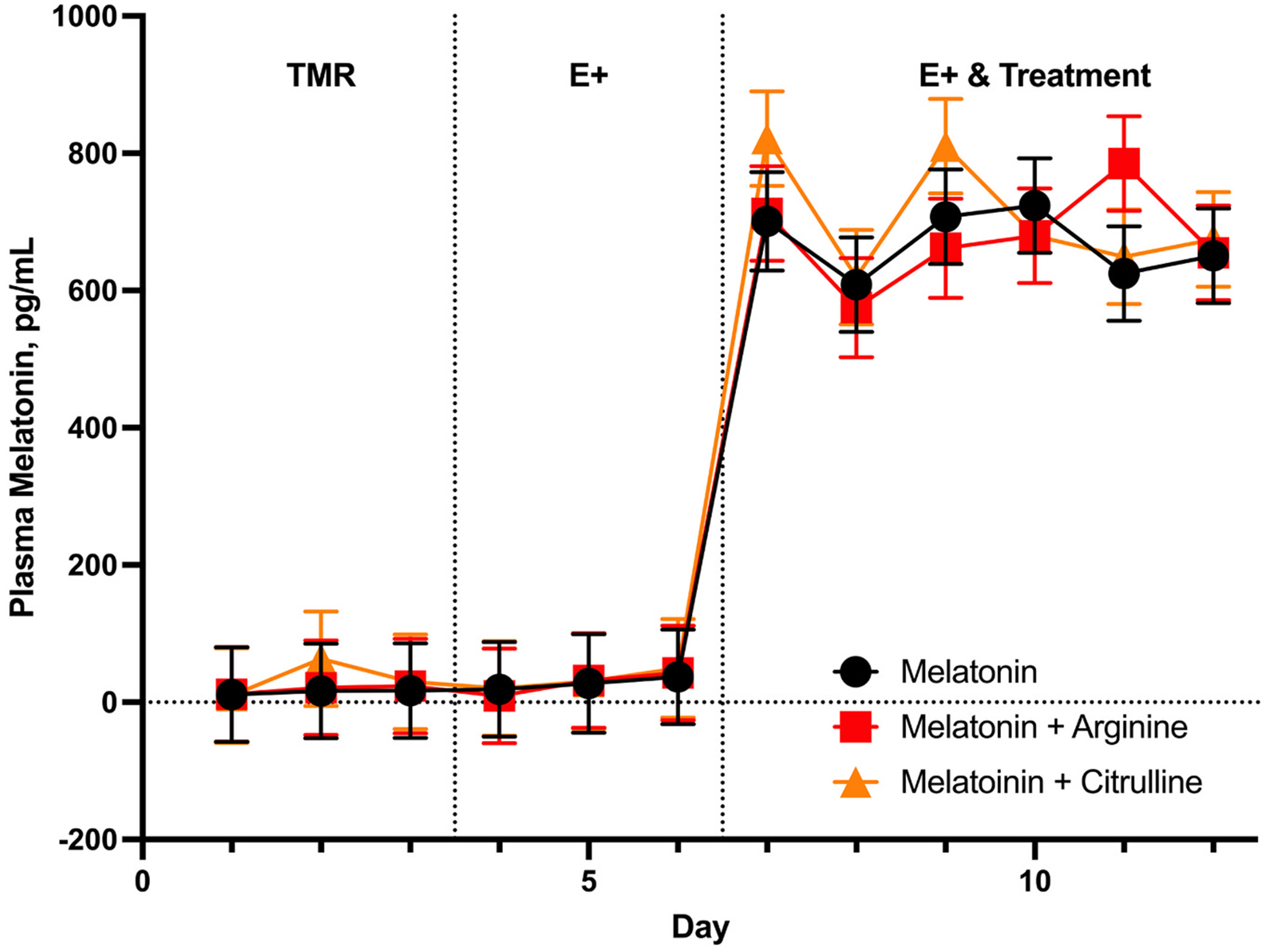
Mean (± SEM) lamb plasma melatonin concentrations for Experiment 2 during the baseline period (d 1–3) where lambs were only consuming a total mixed ration (TMR); d 4–6 where lambs received a daily dose of toxic endophyte-infected tall fescue seed (E + ), and d 7–12 where lambs received the E + and the melatonin or melatonin + amino acid treatments. Melatonin (black circles; n = 6 lambs), melatonin + arginine (red squares; n = 6 lambs), and melatonin + citrulline (orange triangles; n = 6 lambs) treatment effect: P = 0.91; effect of sample day: P < 0.01; and treatment x sample day interaction: P = 0.62.

Experiment 1 confirmed that melatonin could be fed to sheep and produce a rapid post-prandial elevation in plasma melatonin concentrations compared to control lambs (P < 0.05; Fig. 2). There was a rapid elevation of plasma melatonin immediately following the dosing that was followed by a gradual decline. By hour 10 the supplemented lambs were no longer different from control lambs (P > 0.05; Fig. 2). Plasma melatonin supplementation in Experiment 2 increased (P < 0.05) on day 7 when dosing started compared to days 1–6 (Fig. 3). Melatonin concentrations did not differ across treatment groups (P > 0.05), stayed elevated for all three treatment groups, and did not differ across day from day 7–12 (Fig. 3). Consumption of ergot alkaloids (d 4–6) did not affect plasma melatonin concentrations when compared to baseline (d 1–3).

**Fig. 4.**
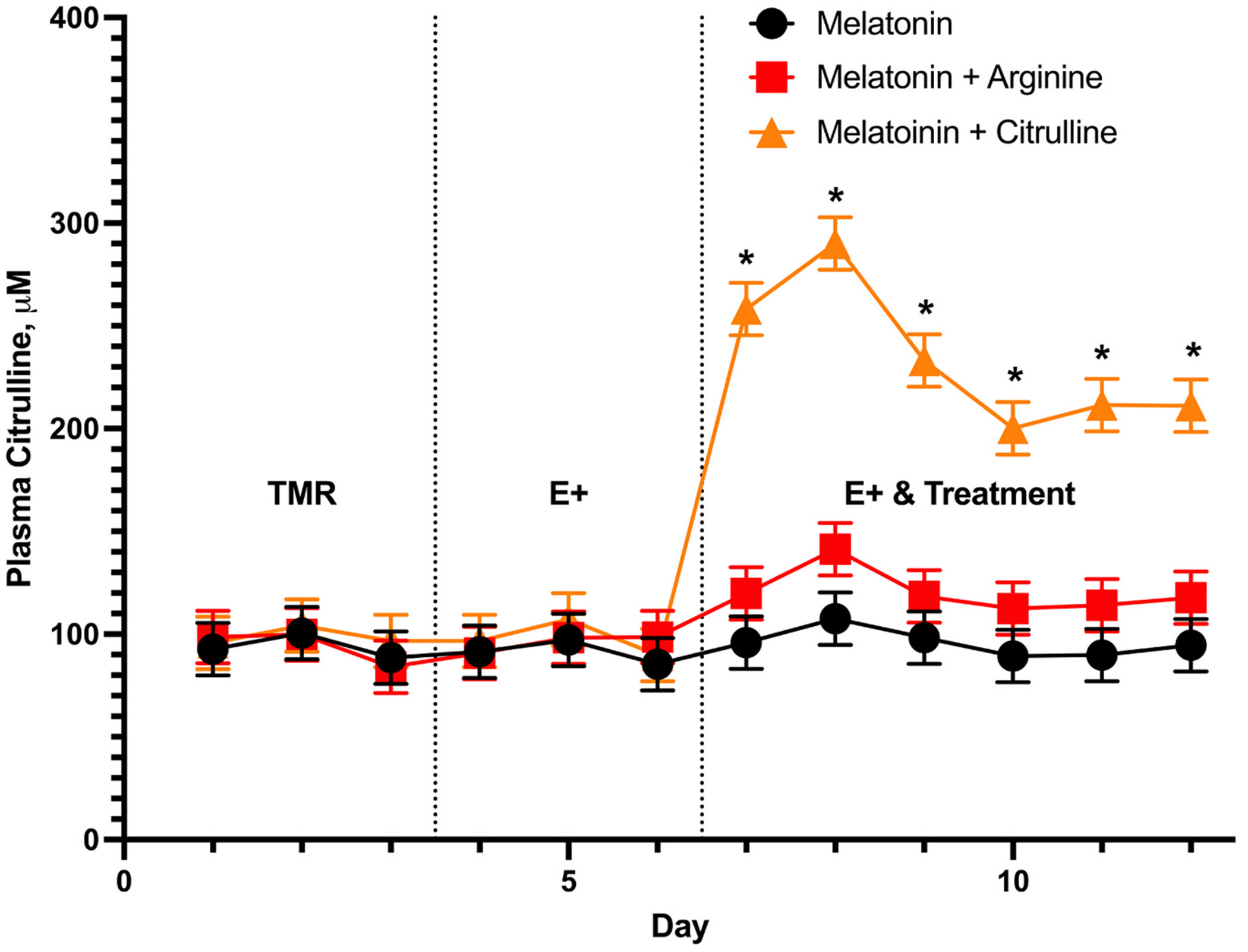
Mean (± SEM) lamb plasma citrulline concentrations for Experiment 2 during the baseline period (d 1–3) where lambs were only consuming a total mixed ration (TMR); d 4–6 where lambs received a daily dose of toxic endophyte-infected tall fescue seed (E + ), and d 7–12 where lambs received the E + and the melatonin or melatonin + amino acid treatments. Melatonin (black circles; n = 6 lambs), melatonin + arginine (red squares; n = 6 lambs), and melatonin + citrulline (orange triangles; n = 6 lambs) treatment effect: P < 0.01; effect of sample day: P < 0.01; and treatment x sample day interaction: P < 0.01. Astericks above data point indicate a significant difference among means within day (P < 0.05).

Melatonin + Citrulline. The plasma concentration of citrulline did not differ between any treatment group during days 1–6 (Fig. 4) prior to dosing. The lambs receiving the melatonin + citrulline treatment had 2- to 3-fold greater plasma citrulline than the melatonin and melatonin + arginine treatment lambs (P < 0.05) during days 7–12. The melatonin + arginine group appeared to have plasma citrulline levels that were slightly and consistently greater than the melatonin only group (P = 0.06; day 8) during the supplementation period. Consumption of ergot alkaloids (d 4–6) did not affect plasma citrulline concentrations when compared to baseline (d 1–3).

**Fig. 5.**
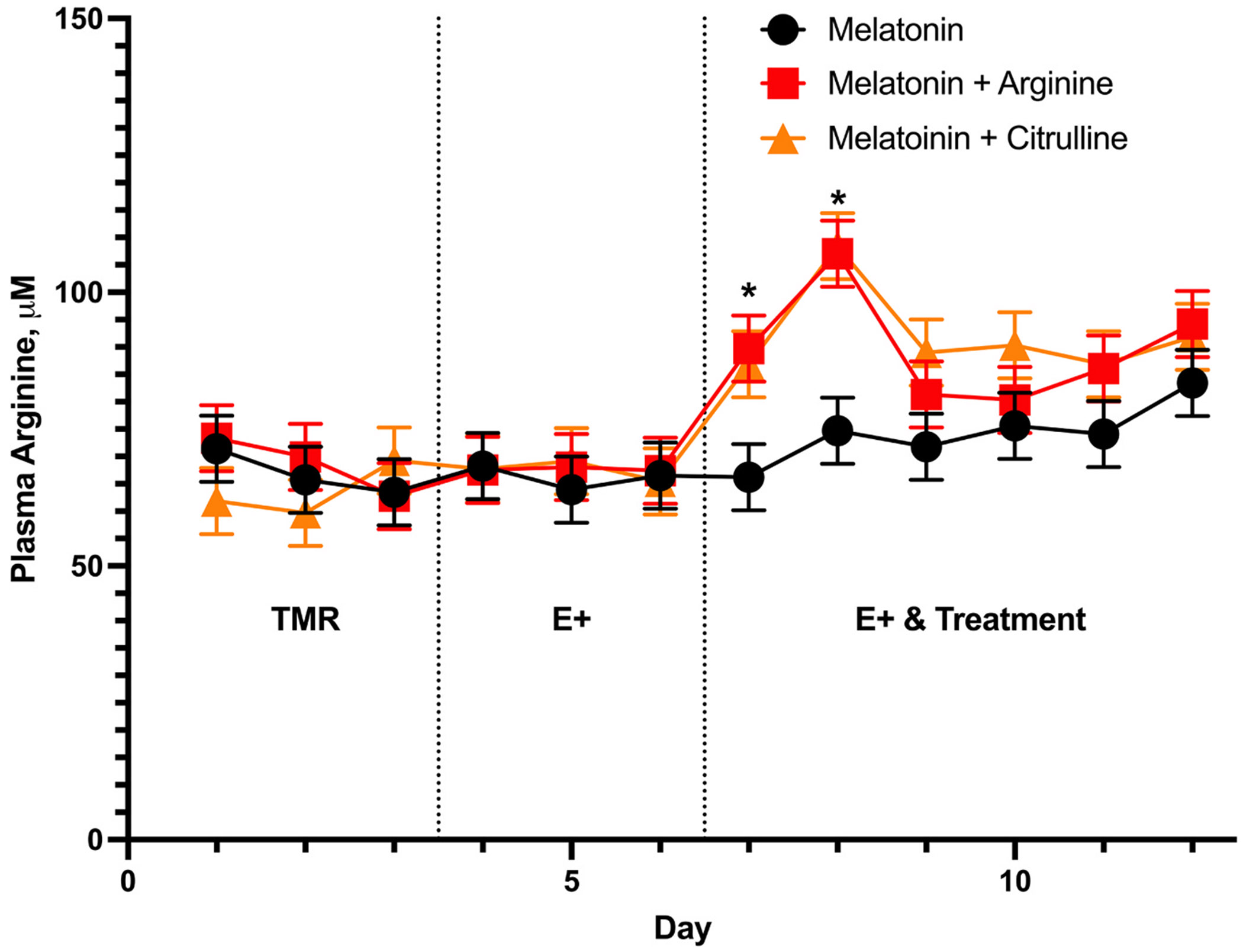
Mean (± SEM) lamb plasma arginine concentrations for Experiment 2 during the baseline period (d 1–3) where lambs were only consuming a total mixed ration (TMR); d 4–6 where lambs received a daily dose of toxic endophyte-infected tall fescue seed (E + ), and d 7–12 where lambs received the E + and the melatonin or melatonin + amino acid treatments. Melatonin (black circles; n = 6 lambs), melatonin + arginine (red squares; n = 6 lambs), and melatonin + citrulline (orange triangles; n = 6 lambs) treatment effect: P = 0.16; effect of sample day: P < 0.01; and treatment x sample day interaction: P = 0.10. Astericks above data point indicate a significant difference among means within day (P < 0.05).

Melatonin + Arginine. The plasma concentrations of arginine did not differ between any treatment groups before the dosing started on day 7 (Fig. 5). On day 7 and 8 the lambs receiving melatonin + citrulline and melatonin + arginine had significantly greater plasma arginine concentrations than the lambs receiving only melatonin (P < 0.05). None of the treatment groups had different plasma arginine levels from day 9–12. Consumption of ergot alkaloids (d 4–6) did not affect plasma arginine concentrations (P > 0.05) when compared to baseline (d 1–3).

### 3.2. Doppler data

**Fig. 6.**
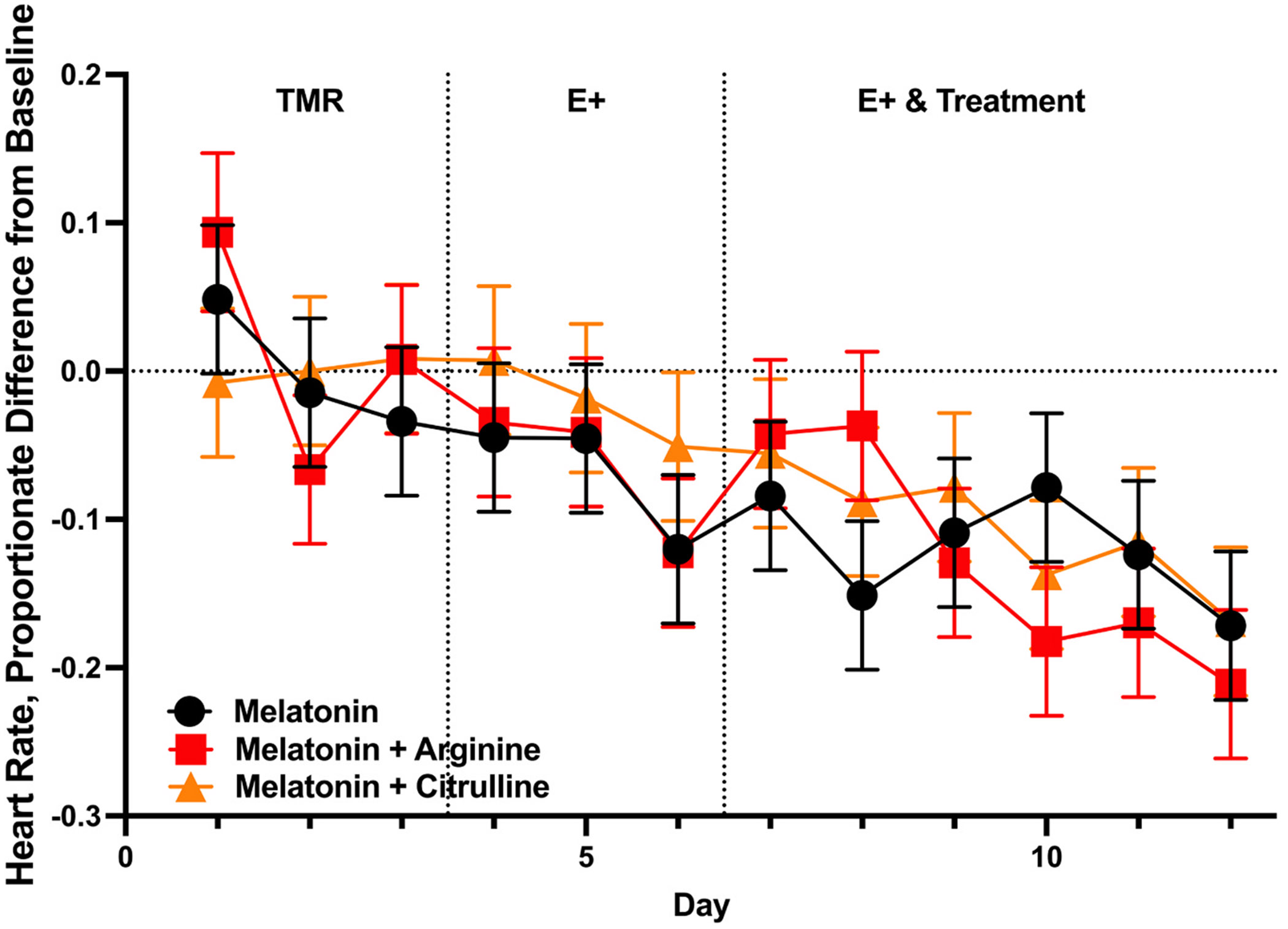
Mean (± SEM) proportionate differences from baseline for lamb heart rates in Experiment 2 during the baseline period (d 1–3) where lambs were only consuming a total mixed ration (TMR); d 4–6 where lambs received a daily dose of toxic endophyte-infected tall fescue seed (E + ), and d 7–12 where lambs received the E + and the melatonin or melatonin + amino acid treatments. Melatonin (black circles; n = 6 lambs), melatonin + arginine (red squares; n = 6 lambs), and melatonin + citrulline (orange triangles; n = 6 lambs) treatment effect: P = 0.88; effect of sample day: P < 0.01; and treatment x sample day interaction: P = 0.68.

**Fig. 7.**
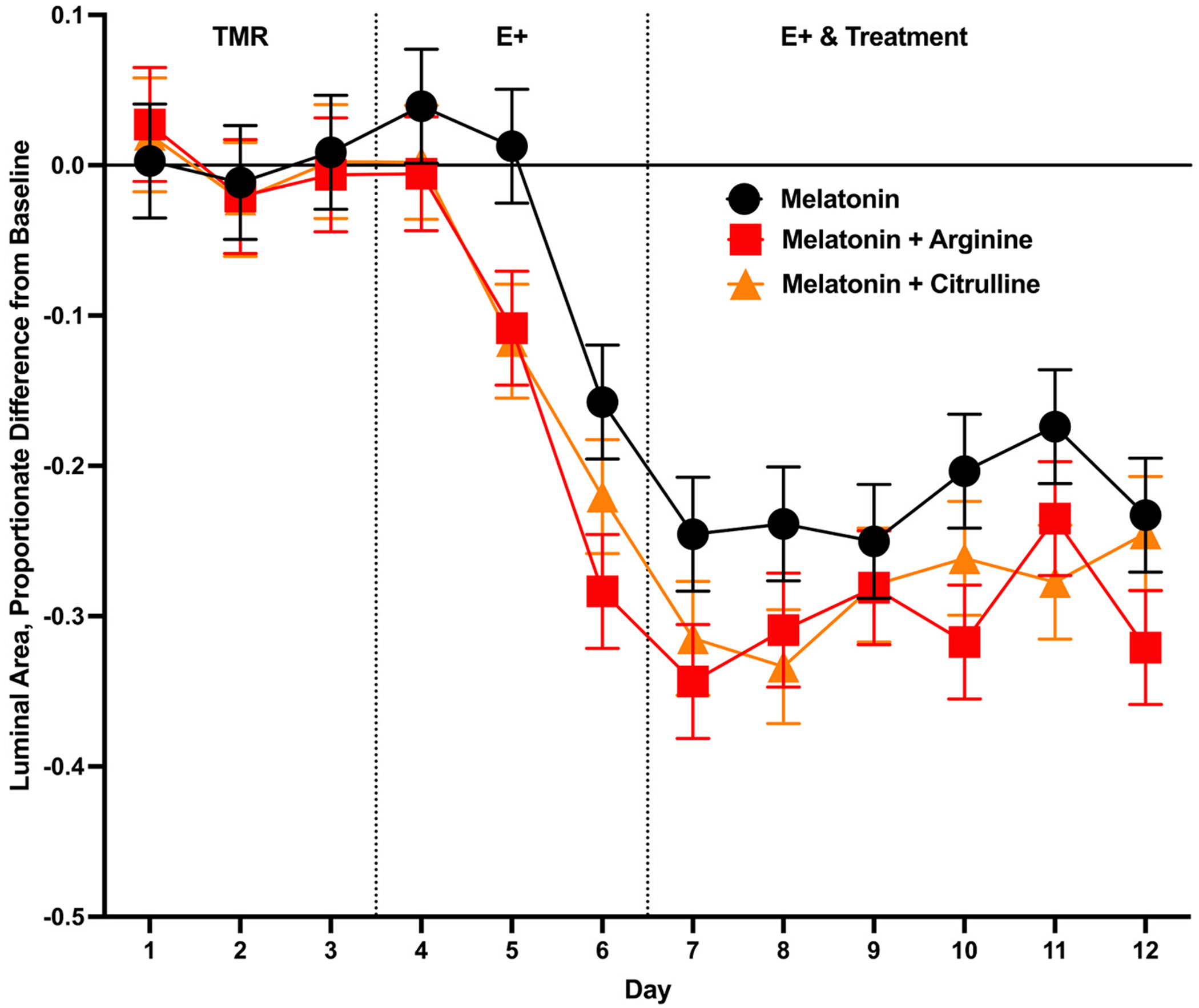
Mean (± SEM) proportionate differences from baseline for luminal area of the carotid artery in Experiment 2 during the baseline period (d 1–3) where lambs were only consuming a total mixed ration (TMR); d 4–6 where lambs received a daily dose of toxic endophyte-infected tall fescue seed (E + ), and d 7–12 where lambs received the E + and the melatonin or melatonin + amino acid treatments. Melatonin (black circles; n = 6 lambs), melatonin + arginine (red squares; n = 6 lambs), and melatonin + citrulline (orange triangles; n = 6 lambs) treatment effect: P = 0.15; effect of sample day: P < 0.01; and treatment x sample day interaction: P = 0.41.

**Fig. 8.**
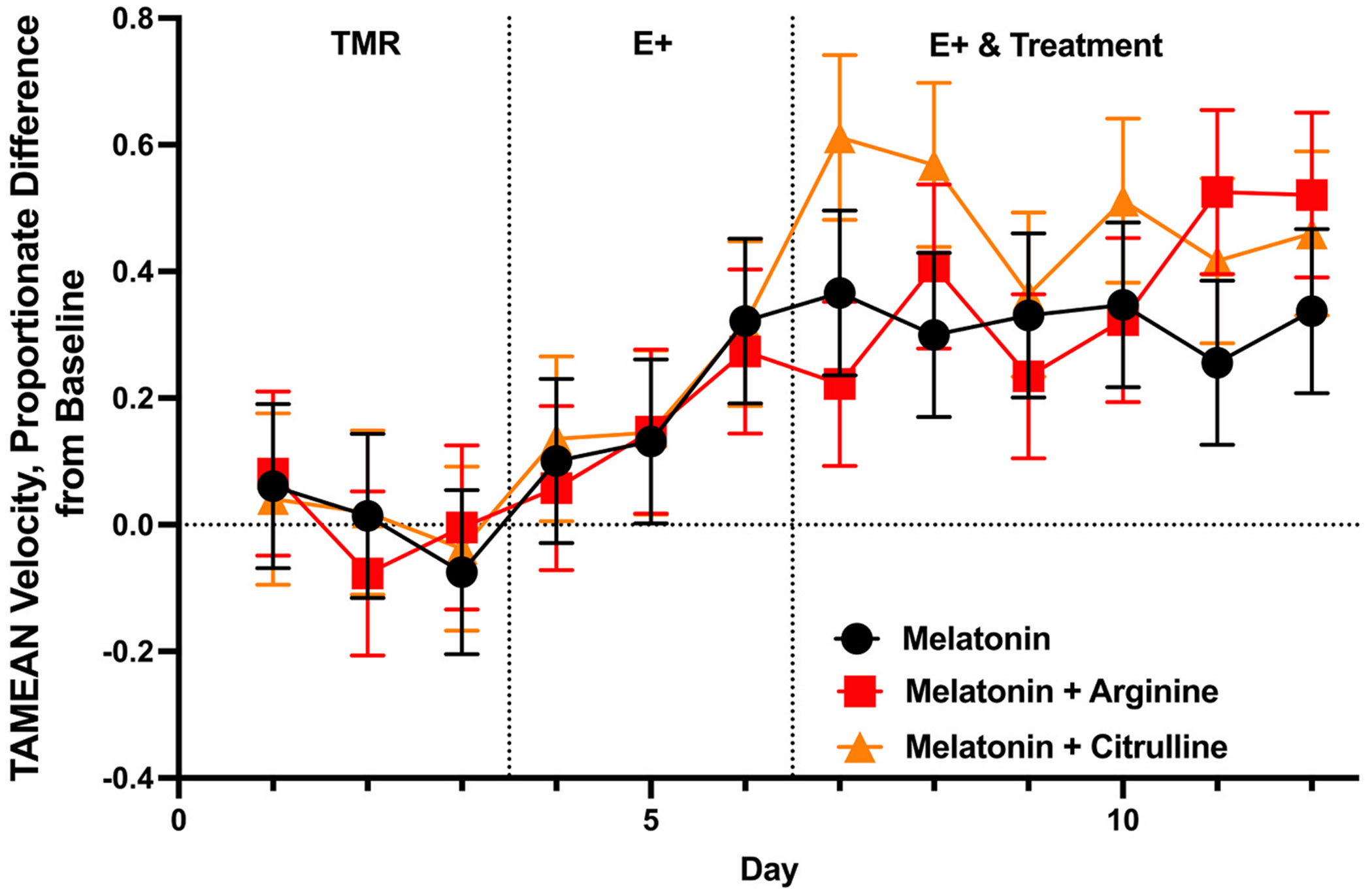
Mean (± SEM) proportionate differences from baseline for time-averaged mean velocity (TAMEAN) of the carotid artery in Experiment 2 during the baseline period (d 1–3) where lambs were only consuming a total mixed ration (TMR); d 4–6 where lambs received a daily dose of toxic endophyte-infected tall fescue seed (E + ), and d 7–12 where lambs received the E + and the melatonin or melatonin + amino acid treatments. Melatonin (black circles; n = 6 lambs), melatonin + arginine (red squares; n = 6 lambs), and melatonin + citrulline (orange triangles; n = 6 lambs) treatment effect: P = 0.76; effect of sample day: P < 0.01; and treatment x sample day interaction: P = 0.69.

**Fig. 9.**
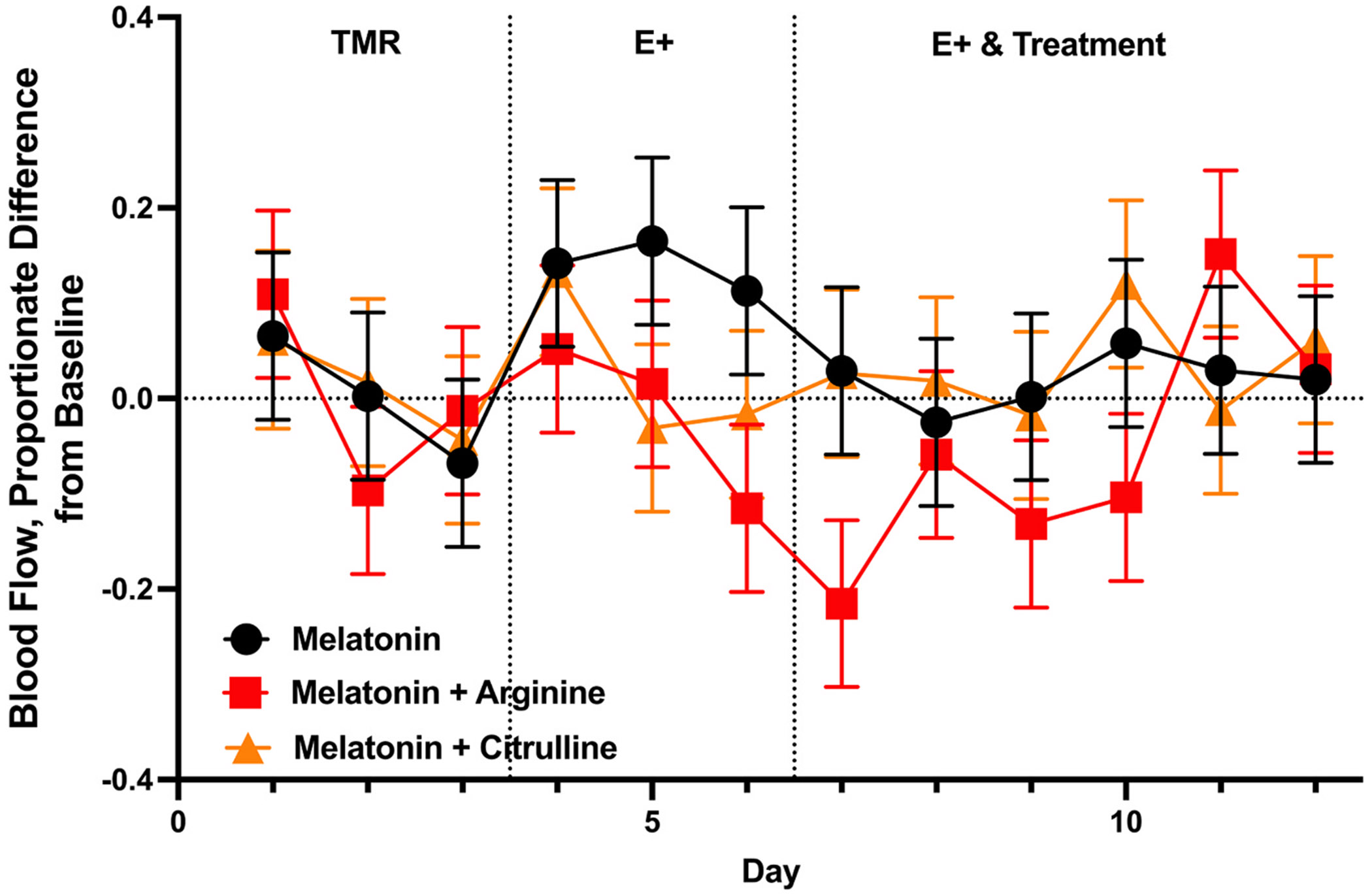
Mean (± SEM) proportionate differences from baseline for calculated volumetric blood flow of the carotid artery in Experiment 2 during the baseline period (d 1–3) where lambs were only consuming a total mixed ration (TMR); d 4–6 where lambs received a daily dose of toxic endophyte-infected tall fescue seed (E + ), and d 7–12 where lambs received the E + and the melatonin or melatonin + amino acid treatments. Melatonin (black circles; n = 6 lambs), melatonin + arginine (red squares; n = 6 lambs), and melatonin + citrulline (orange triangles; n = 6 lambs) treatment effect: P = 0.61; effect of sample day: P = 0.09; and treatment x sample day interaction: P = 0.36.

Effect of sample day was significant with heart rate measurements with a steady decline associated with the start of the E + ergot alkaloids on d 4 (P < 0.01; Fig. 6). This decline was not mitigated by the start of the treatments on d7 as the heart rate continued to decline through to d 12 for all 3 treatments (P = 0.68). There was a precipitous decline in luminal area of the carotid artery associated with the start of the E + ergot alkaloids on d4 that plateaued starting on d 7 (P < 0.01; Fig. 7). There was no difference induced by the inclusion of MEL, MEL+ARG, or MEL+CIT on off-setting the ergot alkaloid-induced decrease in arterial luminal area (P = 0.41). Conversely, the time-averaged mean velocity of blood flowing through the carotid artery increased in association with the start of the E + ergot alkaloids (P < 0.01; Fig. 8). There was no difference in velocity associated the start of any of the mitigation treatments from d 7 through d 12 (P = 0.69). The calculated blood flow tended to differ by day (P = 0.09; Fig. 9); however, there was no interaction with day and mitigation treatments (P = 0.36).

## 4. Discussion

The goal of the experiments in this study was to determine if the use of the hormone melatonin alone or in combination with the amnio acids arginine and citrulline could be used as effective supplements to mitigate the negative vascular effects of fescue toxicosis. Elevated circulating concentrations of melatonin and citrulline were achieved with the supplementation of these compounds. Vasoconstriction of the carotid artery with E + fescue seed was also achieved as measured through a decreased luminal area; however, the elevated levels of MEL and MEL+CIT were not sufficient to counteract the ergot alkaloid-induced vasoconstriction at the carotid artery level.

Melatonin has previously been shown to play a regulatory role in the cardiovascular system (Mahle et al., 1997). Specifically, melatonin has been shown to mediate vasodilation in a variety of blood vessels and species, such as the rat aorta and mesenteric arteries (Girouard et al., 2001; Zhao et al., 2017), sheep umbilical artery (Thakor et al., 2010), bovine uterine artery (Contreras-Correa et al., 2021) for example. Supplementation of arginine and citrulline are effective at increasing circulating arginine which has been shown to influence cardiovascular tone (Khalaf et al., 2019). This is accomplished by arginine’s role in the generation of NO (Wu and Morris, 1998). Nitric oxide has been shown to act as a relaxant derived from endothelial cells (Palmer et al., 1988). The interaction of melatonin with the MT2 receptor can also stimulate endothelial NO production and correspondingly stimulate vasorelaxation (Paulis and Simko, 2007; Reiter et al., 2009).

The basic premise of this study’s hypothesis was that melatonin, melatonin + citrulline, or melatonin + arginine would stimulate vasorelaxation as a result of a corresponding elevated NO release in blood vessels. Nitric oxide stimulates guanylyl cyclase in the vascular smooth muscle layer of a blood vessel that results in elevated production of cyclic guanosine monophosphate (cGMP). The increase in cGMP results in the activation of protein kinase G. This enzyme has several functions that include promotion of calcium reuptake by the sarcoplasmic reticulum through the activation of the local calcium ATPase and prevents inositol 1,4,5-triphosphate derived calcium release. This decrease in cytosolic calcium results in the inactivation of myosin light chain kinase and the corresponding phosphorylation of myosin decreases. The PKG-derived decrease in intracellular calcium also increases the activity of myosin light chain phosphatases that inactivate the myosin light chains which are involved in vascular smooth muscle contraction and ultimately result in NO-derived vasorelaxation (Zhao et al., 2015). Green et al. (2017) evaluated the use of rumen protected arginine in beef cows consuming toxic endophyte-infected tall fescue (E + ) and showed that supplementing arginine at 180 mg/kg BW increased circulating NO concentrations in the E + cows but not the control cows. Green et al. (2017) reported vasoconstriction of the caudal artery due to consumption of toxic endophyte-infected tall fescue seed and this decrease in luminal area did not change in response to the supplementation of arginine. This is similar to the current study, where the level of ergot alkaloid administration was sufficient to induce vasoconstriction of the carotid artery with a significant decrease in luminal area and like Green et al. (2017) this constriction was not altered with either the arginine or citrulline treatments. Similarly, Snider et al. (2024) supplemented melatonin to multiparous cows also receiving of toxic endophyte-infected tall fescue seed. There was a vasoconstrictive effect of the ergot alkaloids in the uterine and coccygeal artery reported, but like the current study there was no mitigation due to the supplementation of melatonin. There was a plateau in the luminal area that coincided with the start of the MEL, MEL+CIT, and MEL+ARG treatments. It is possible that the treatments prevented further vasoconstriction caused by the ergot alkaloids in the E + tall fescue. Because of the experimental design of this study, there was no E + fescue only control. This would have afforded the observation of a continued decrease in luminal area of the carotid artery in the absence of a mitigation treatment. Future work will be needed to confirm this.

The experimental design incorporating an ergot alkaloid-induced vasoconstriction to evaluate a compound for vasorelaxation has been shown previously to be effective for isoflavones producing vasorelaxation in the carotid artery of goats that was ergot alkaloid constricted (Aiken et al., 2016). Previous work with the inclusion of a melatonin supplement at the dosage similar to or less than the dosage used in the current study has been shown to produce increased umbilical artery blood flow in nutrient restricted lambs (Lemley et al., 2012, 2013). Although, it is possible that the length of time that the melatonin treatment was administered was not sufficient as Lemley et al. (2012) reported differences in blood flow due to melatonin after 10 d of administration. However, Snider et al. (2024) supplemented melatonin at the same dosage as the current study for longer than 10 d and did not report a mitigation of the ergot alkaloid-induced vasoconstriction. Similarly, Green et al. (2017) reported an increase in NO in cattle associated with arginine supplementation at a 180 mg/kg BW compared to 81 mg/kg BW for citrulline and arginine in the current study. The dose for citrulline and arginine used in the current study was based on previous work (Lassala et al., 2009, 2011; Wu et al., 2016; Greene et al., 2020b). The NO concentration necessary to offset ergot alkaloid-induced vasoconstriction is presently unknown. Future work will have to determine if increased dosages of melatonin, citrulline, and arginine and extended time that treatments were administered could have resulted in a different outcome.

In conclusion, supplementation of melatonin and citrulline successfully elevated circulating concentrations of these compounds, whereas supplementation of arginine and citrulline only caused a minor elevation in blood arginine concentrations. The supplementation of toxic endophyte-infected tall fescue seed did have the desired effect of causing vasoconstriction as evidenced by the decreased carotid artery luminal area and increased velocity. The supplementation of melatonin or melatonin plus citrulline or arginine did not offset the negative vascular effects of the ergot alkaloids in the toxic tall fescue seed. Further work looking at the supplementation of these nitric oxide-generating compounds should evaluate elevated doses for extended periods of time to generate sufficient nitric oxide to produce vasorelaxation necessary to mitigate the negative vascular effects of fescue toxicosis.

## Figures and Tables

**Table 1:** Composition of total mixed ration (TMR) fed to ewe lambs in Experiments 1 and 2.

Ingredient	% of ration, DM basis
Cottonseed hulls	25
Molasses	14
Corn, cracked	35
Soybean hulls	20.5
Limestone	1.0
Soybean meal	4.5
**Nutrient Composition**	**kg/hd/d DM**
Dry matter	1.03
TDN	0.71
Crude protein	0.125

## References

[R1] Aiken GE, Flythe MD, Kagan IA, Ji H, Bush LP, 2016. Mitigation of ergot vasoconstriction by clover isoflavones in goats (Capra hircus). Front. Vet. Sci 3, 17. 10.3389/fvets.2016.00017. 26973844 PMC4777723

[R2] Berde B, 1980. Ergot compounds: a synopsis. Raven Press, New York, NY. 6104909

[R3] Blanco P, 2015. Volumetric blood flow measurement using Doppler ultrasound: concerns about the technique. J. Ultrasound 18 (2), 201–204. 10.1007/s40477-015-0164-3. 26191112 PMC4504867

[R4] Bode-Boger SM, Boger RH, Galland A, Tsikas D, Frolich JC, 1998. Br. J. Clin. Pharm465489497 10.1046/j.1365-2125.1998.00803.x. PMC1873701 9833603

[R5] Chalupa W, 1976. Degradation of amino acids by the mixed rumen microbial population. J. Anim. Sci 43 (4), 828–834. 10.2527/jas1976.434828x. 977500

[R6] Contreras-Correa ZE, Messman RD, Sidelinger DR, Heath King E, Sanchez-Rodriguez HL, Burnett DD, Lemley CO, 2021. Melatonin alters bovine uterine artery hemodynamics, vaginal temperatures, and fetal morphometrics during late gestational nutrient restriction in a season-dependent manner. J. Anim. Sci 99 (9). 10.1093/jas/skab242. PMC8420683 34387666

[R7] Das R, Balonan L, Ballard HJ, Ho S, 2008. Chronic hypoxia inhibits the antihypertensive effect of melatonin on pulmonary artery. Int. J. Cardiol 126 (3), 340–345. 10.1016/j.ijcard.2007.04.030. 17590454

[R8] Gilbreath KR, Bazer FW, Satterfield MC, Cleere JJ, Wu G, 2020a. Ruminal microbes of adult sheep do not degrade extracellular l-citrulline. J. Anim. Sci 98 (6). 10.1093/jas/skaa164. PMC7344112 32415842

[R9] Gilbreath KR, Nawaratna GI, Wickersham TA, Satterfield MC, Bazer FW, Wu G, 2020b. Metabolic studies reveal that ruminal microbes of adult steers do not degrade rumen-protected or unprotected L-citrulline. J. Anim. Sci 98 (1). 10.1093/jas/skz370. PMC6986429 31830257

[R10] Girouard H, Chulak C, Lejossec M, Lamontagne D, de Champlain J, 2001. Vasorelaxant effects of the chronic treatment with melatonin on mesenteric artery and aorta of spontaneously hypertensive rats. J. Hypertens 19 (8), 1369–1377. 11518844 10.1097/00004872-200108000-00004

[R11] Green MA, Whitlock BK, Edwards JL, Scholljegerdes EJ, Mulliniks JT, 2017. Rumen-protected arginine alters blood flow parameters and luteinizing hormone concentration in cyclic beef cows consuming toxic endophyte-infected tall fescue seed. J. Anim. Sci 95 (4), 1537–1544. 10.2527/jas.2016.1309. 28464107

[R12] Greene MA, Britt JL, Bertrand JK, Klotz JL, Bridges W Jr., Andrae JG, Duckett SK, 2020a. Feeding tall fescue seed during mid and late gestation influences subsequent postnatal growth, puberty, and carcass quality of offspring. Animals 10 (10). 10.3390/ani10101859. PMC7601090 33053893

[R13] Greene MA, Klotz JL, Goodman JP, May JB, Harlow BE, Baldwin WS, Strickland JR, Britt JL, Schrick FN, Duckett SK, 2020b. Evaluation of oral citrulline administration as a mitigation strategy for fescue toxicosis in sheep. Transl. Anim. Sci 4 (4), txaa197. 10.1093/tas/txaa197. 33269340 PMC7684870

[R14] Khalaf D, Kruger M, Wehland M, Infanger M, Grimm D, 2019. The effects of oral l-Arginine and l-Citrulline supplementation on blood pressure. Nutrients 11 (7). 10.3390/nu11071679. PMC6683098 31336573

[R15] Klotz JL, 2015. Activities and effects of ergot alkaloids on livestock physiology and production. Toxins 7 (8), 2801–2821. 10.3390/toxins7082801. 26226000 PMC4549725

[R16] Lassala A, Bazer FW, Cudd TA, Li P, Li X, Satterfield MC, Spencer TE, Wu G, 2009. Intravenous administration of L-citrulline to pregnant ewes is more effective than L-arginine for increasing arginine availability in the fetus. J. Nutr 139 (4), 660–665. 10.3945/jn.108.102020. 19225132 PMC2666359

[R17] Lassala A, Bazer FW, Cudd TA, Datta S, Keisler DH, Satterfield MC, Spencer TE, Wu G, 2011. Parenteral administration of L-arginine enhances fetal survival and growth in sheep carrying multiple fetuses. J. Nutr 141 (5), 849–855. 10.3945/jn.111.138172. 21430253 PMC3078019

[R18] Lemley CO, Camacho LE, Meyer AM, Kapphahn M, Caton JS, Vonnahme KA, 2013. Dietary melatonin supplementation alters uteroplacental amino acid flux during intrauterine growth restriction in ewes. Animal 7 (9), 1500–1507. 10.1017/S1751731113001006. 23764235

[R19] Lemley CO, Meyer AM, Camacho LE, Neville TL, Newman DJ, Caton JS, Vonnahme KA, 2012. Melatonin supplementation alters uteroplacental hemodynamics and fetal development in an ovine model of intrauterine growth restriction. Am. J. Physiol. Regul. Integr. Comp. Physiol 302 (4), R454–R467. 10.1152/ajpregu.00407.2011. 22129617

[R20] Lemley CO, Vonnahme KA, 2017. Physiology and endocrinology symposium: alterations in uteroplacental hemodynamics during melatonin supplementation in sheep and cattle. J. Anim. Sci 95 (5). 10.2527/jas2016.1151. 28726984

[R21] Lorente JA, Landin L, Renes E, Pablo R.De, Jorge P, Rodena E, Liste D, 1993. Role of nitric oxide in the hemodynamic changes of sepsis. Crit. Care Med 21 (5), 759–767. 10.1097/00003246-199305000-00021. 8482098

[R22] Mahle CD, Goggins GD, Agarwal P, Ryan E, Watson AJ, 1997. Melatonin modulates vascular smooth muscle tone. J. Biol. Rhythms 12 (6), 690–696. 9406046 10.1177/074873049701200626

[R23] Palmer RM, Ashton DS, Moncada S, 1988. Vascular endothelial cells synthesize nitric oxide from L-arginine. Nature 333 (6174), 664–666. 10.1038/333664a0. 3131684

[R24] Paulis L, Simko F, 2007. Blood pressure modulation and cardiovascular protection by melatonin: potential mechanisms behind. Physiol. Res 56 (6), 671–684. 10.33549/physiolres.931236. 18197748

[R25] Reiter RJ, Tan DX, Korkmaz A, 2009. The circadian melatonin rhythm and its modulation: possible impact on hypertension. J. Hypertens. Suppl 27 (6), S17–S20. 10.1097/01.hjh.0000358832.41181.bf. 19633446

[R26] Snider MA, Culp TC, Hopkins CR, Moss NT, Edwards JL, Looney CR, Powell JG, Kegley EB, Coffey KP, Gadberry MS, Lemley CO, Littlejohn BP, 2024. 232 influence of melatonin supplementation on uterine and coccygeal artery hemodynamics of pregnant multiparous crossbred beef cows grazing endophyte-infected tall fescue. J. Anim. Sci 102 (3), 284–285. 10.1093/jas/skae234.326.

[R27] Strickland JR, Looper ML, Matthews JC, Rosenkrans CF Jr., Flythe MD, Brown KR, 2011. Board-invited review: st. Anthony’s fire in livestock: causes, mechanisms, and potential solutions. J. Anim. Sci 89 (5), 1603–1626. 10.2527/jas.2010-3478. 21521821

[R28] Thakor AS, Herrera EA, Seron-Ferre M, Giussani DA, 2010. Melatonin and vitamin c increase umbilical blood flow via nitric oxide-dependent mechanisms. J. Pineal Res 49 (4), 399–406. 10.1111/j.1600-079X.2010.00813.x. 20958954

[R29] Trotta RJ, Lemley CO, Vonnahme KA, Swanson KC, 2021. Effects of nutrient restriction and melatonin supplementation from mid-to-late gestation on maternal and fetal small intestinal carbohydrase activities in sheep. Domest. Anim. Endocrinol 74, 106555. 10.1016/j.domaniend.2020.106555. 32947201

[R30] Wu Z, Hou Y, Hu S, Bazer FW, Meininger CJ, McNeal CJ, Wu G, 2016. Catabolism and safety of supplemental L-arginine in animals. Amino Acids 48 (7), 1541–1552. 10.1007/s00726-016-2245-9. 27156062

[R31] Wu G, Morris SM Jr., 1998. Arginine metabolism: nitric oxide and beyond. Biochem. J 336 (Pt1), 1–17. 10.1042/bj3360001. 9806879 PMC1219836

[R32] Zhao Y, Vanhoutte PM, Leung SW, 2015. Vascular nitric oxide: beyond eNOS. J. Pharm. Sci 129 (2), 83–94. 10.1016/j.jphs.2015.09.002. 26499181

[R33] Zhao H, Wang Y, Jin Y, Liu S, Xu H, Lu X, 2016. Rapid and sensitive analysis of melatonin by LC-MS/MS and its application to pharmacokinetic study in dogs. Asian J. Pharm. Sci 11 (2), 273–280. 10.1016/j.ajps.2015.08.004.

[R34] Zhao T, Zhang H, Jin C, Qiu F, Wu Y, Shi L, 2017. Melatonin mediates vasodilation through both direct and indirect activation of BK(Ca) channels. J. Mol. Endocrinol 59 (3), 219–233. 10.1530/JME-17-0028. 28676563

